# Progression of Large Lymphoma Is Significantly Impeded with a Combination of Gemcitabine Chemotherapy and Dendritic Cells Intra-Tumor Vaccination

**DOI:** 10.1371/journal.pone.0132799

**Published:** 2015-07-16

**Authors:** Xue-Jun Zhu, Zhong-Fa Yang, Jin-Yong Zhou, Li Liu, Xue-Mei Sun, Zhen-Fang Fan, Shou-You Hu, Yu-Chao Chen, Wei-Xia Li, Meng Cao, Li-Xin Wang

**Affiliations:** 1 Department of Microbiology and Immunology, Medical School of Southeast University, Nanjing, Jiangsu Province, People’s Republic of China; 2 Division of Hematology, Department of Medicine, Jiangsu Provincial Traditional Chinese Medical Hospital, Nanjing, Jiangsu Province, People’s Republic of China; 3 Division of Hematology-Oncology, Department of Medicine, University of Massachusetts Medical School, Worcester, Massachusetts, United States of America; Mie University Graduate School of Medicine, JAPAN

## Abstract

Relapsed, refractory lymphoma remains to be a challenge and lacks efficient treatment. Some tumor cells escape from treatment, become resistant to chemotherapeutic agents, and rapidly regenerate into large tumors. Lymphoma cells induce accumulation of Gr-1^+^CD11b^+^ myeloid derived suppressor cells (MDSCs) in lymphatic organs and their vicinity. MDSCs enable tumor cells to escape from immune cells mediated surveillance and attack. Gemcitabine is a chemotherapeutic agent that eliminates both tumor cells and MDSCs, improving the immune environment favorable for subsequent treatment. We evaluated the effects of low dose gemcitabine combined with intra-tumorally delivered dendritic cells (DCs) for the treatment of A20 large-size lymphoma. We showed that MDSCs increased markedly in lymphoma-bearing mice, and that gemcitabine significantly increased the apoptosis of MDSCs. Treatment of lymphoma with either gemcitabine or intra-tumoral DCs alone could not inhibit tumor growth or rescue lymphoma-bearing mice. Treatment of lymphoma with small dose gemcitabine followed by intra-tumorally injected DCs significantly improved the efficacy of either individual treatment by reducing MDSCs, inducing onsite DCs maturation, eliminating tumor cells, inhibiting tumor growth and relapse, and extending the survival of the lymphoma-bearing mice, partly through the induction of the IFNγ secreting cells and the activation of cytotoxic lymphocytes. We showed that NK cells and CD8^+^ T cells were the major effectors to mediate the inhibition of tumor growth. Thus, the observation that gemcitabine synergizes DCs mediated immunotherapy to improve the efficacy of large size lymphoma treatment provides an experimental basis for the combination of chemotherapy and immunotherapy for the efficient treatment of relapsed or refractory lymphoma.

## Introduction

Lymphomas represent the fourth most common hematologic malignancy among western countries [1]. They are highly heterogeneous diseases, varying by both the type of malignant cell and the tumor location. Nowadays chemotherapy is the major option for treatment of both Hodgkin's and non-Hodgkin's lymphomas [2]. For large size lymphomas, treatment with deliberately designed chemotherapeutic regime can efficiently inhibit tumor growth and eliminate the majority of tumor cells. However, a small number of residual tumor cells that manage to escape from chemotherapeutic treatment become resistant or unresponsive to the original treatment. These relapsed or refractory lymphomas still remain a challenge and lack efficient clinical treatment. Thus, novel strategies are required to develop for the treatment of relapsed or refractory lymphomas.

Tumor cells can be recognized by tumor-specific T cells [3]. Moreover, tumor infiltration with cytotoxic T lymphocytes (CTLs) and T helper cells represents a favorable prognostic factor for lymphoma patients. Tumor-specific T cells can be activated by vaccination with dendritic cells (DCs) [4,5,6]. DCs are unique antigen-presenting cells that deliver exogenous antigens into the major histocompatibility complex (MHC) class I processing pathway to activate CTLs [7]. Contact with microbial, inflammatory, and T cell-derived activation signals induces DC maturation and secretion of cytokine molecules, which in turn activate CTLs, natural killer (NK) cells, and interferon gamma (IFNγ)-producing T helper type 1 (Th1) cells. Vaccination with in vitro pulsed, tumor antigen-loaded DCs has been shown to elicit anti-tumor CTL responses and to induce tumor regression in cancer patients [8]. However, a number of recent clinical trials of vaccination with lymphoma antigen-activated DCs have failed to demonstrate the expected, desired results [9]. One of the reasons is that tumor antigen pulsed, *in vitro* matured DCs may not be as efficient as *in vivo* matured DCs in either function or quantity. Another possible reason is that relapsed, refractory lymphomas that resistant to chemotherapeutic agents often form large size tumors, but Immune cancer therapy with DCs is more efficient to eliminate small size tumors rather than tumors with large size [10,11]. Finally, it is proposed that tumors can form an immunosuppressive environment rendering them insensitive to T cells and NK cells [12,13]. Thus, the improvement of the current DC based immunotherapy and the combination of multiple strategies, including radiation therapy, chemotherapy and other immunotherapy, are required to achieve more efficient treatment of the large lymphoma in patients.

Myeloid derived suppressor cells (MDSCs) are a population of cells derived from the myeloid lineages that can account for 10–40% of spleen nucleated cells of tumor-bearing animals [14]. These cells had the ability to inhibit T cell proliferation, to promote tumor growth, and to suppress graft-versus-host disease (GVHD) [15]. Young et al first described the accumulation of a large number of myeloid suppressor cells around tumor tissues of patients with head and neck cancer and renal cell carcinoma patients [16]. In mouse the characteristic immunophenotype of MDSCs is the expression of myeloid cell surface markers, CD11b and Gr-1 [17]. In human MDSCs their cell surface immunophenotype is CD11b^+^CD15^+^CD33^+^CD13^+^ CD34^+^CD14^-^HLA^-^DR^-^ [18]. Through the production of nitric oxide (NO) and L-arginine (ARG1), MDSCs from tumor-bearing animals suppress the expression of the CD3ζ chain of the T-cell receptor and L-Selectin, inhibit antigen-specific responses from CD8^+^ T cells, induce to generate regulatory T cells, IL-7 and IL-15, and inhibit the NK cells and the cytotoxic activity of NKT cells [19,20,21,22,23,24,25,26]. Given these immunosuppressive effects, it has been proposed that elimination of these myeloid suppressor cells may significantly improve anti-tumor responses and enhance effects of cancer immunotherapy [17]. Kusmartsev et al. reported that all-trans retinoic acid (ATRA) was able to induce differentiation of CD11b^+^Gr-1^+^ MDSCs, and thus reduced immunosuppression and improved the effect of a tumor vaccine in a mouse model [27].

Gemcitabine, the nucleoside analog chemotherapeutic agent, not only exerts direct anti-tumor activity, but also mediates tumor immunotherapeutic effects. Gemcitabine has been shown to selectively reduce the number of CD11b^+^Gr-1^+^ cells and B cells, while preserving CD4^+^ T cells and CD8^+^ T cells in the spleens of mice bearing murine mesotheliomas [28,29]. Gemcitabine-mediated reduction in MDSCs resulted in the amplification of CTLs, increased activity of antitumor CD8^+^ T cells and NK cells, and led to the increase of immunogene-therapy using an adenoviral-expressing IFN-β to treat large tumors [30]. Moreover, Bauer et al showed that gemcitabine combined with DC-based vaccination increase survival in a mouse model of pancreatic cancer, and prophylactic DC vaccination can prevent metastatisation and orthotopic growth of murine pancreatic carcinoma cells [31].

In this report, we used A20 cells to establish a large size, murine B-cell lymphoma model. We confirmed the accumulation of MDSCs in the spleen of lymphoma-bearing mice. Gemcitabine induced increased apoptosis in both cultured MDSCs and A20 cells. Injection of gemcitabine eliminated the majority of MDSCs in the spleen of lymphoma-bearing mice, and produced lymphoma damage-associated molecular-pattern molecules (DAMPs) for in vivo DC activation. Gemcitabine treatment combined with intra-tumoral injection of inactivated DCs promoted the onsite DC maturation, induced the replication and activation of the cytokine secreting NK cells and T lymphocytes, which in turn markedly reduced the size of tumor, and extended the survival of the lymphoma-bearing mice. We demonstrated that intra-large-size-lymphoma injection with DCs in combination with gemcitabine chemotherapy enhanced the therapeutic efficacy of either DC-based vaccination or chemotherapeutic reagent alone. Together with other studies, our report supports the hypothesis that gemcitabine-mediated reduction in MDSCs significantly reduces the immune suppression in tumor-bearing animal environment. This study established novel experimental foundation for the combination of the improved immune-chemotherapy to treat relapsed or refractory lymphoma in large size with enhanced efficacy.

## Materials and Methods

### Mice

Six to eight week old male BALB/c (H-2^d^) mice were purchased from the Center of Comparative Medical Animals of Yangzhou University (Yangzhou, Jiangsu). Animal studies were performed in strict accordance with the recommendations in the Guide for the Care and Use of Laboratory Animals of the Jiangsu Provincial Traditional Chinese Medical Hospital (Nanjing, Jiangsu). The protocol was approved by the Committee on the Ethics of Animal Experiments of the Jiangsu Provincial Traditional Chinese Medical Hospital (Permit Number CN-JS-2007). Most mice died of the direct result of the lymphoma within the designated endpoint period (120 days). Mice with certain treatment that survived beyond the endpoint period, or mice developed tumors larger than 2800 mm^3^, or mice extremely suffered from the tumors and expected to die within the same day of observation, as judged by monitoring the abnormality of mice including lack of eating/drinking/moving activity under mild stimulation, shivering, eye lids shut etc, were humanely euthanized on the days of observation. We monitored the mice daily for survival studies. Mice that survived beyond the satisfied experimental designed period (120 days) were humanely euthanized with CO_2_ followed by cervical dislocation. Mice survival and death were monitored daily until their death or the experimental designed endpoint. All surgery was performed under sodium pentobarbital anesthesia, and every effort was made to minimize suffering.

### A20 lymphoma cell line and mouse DC cell culture

A20 B cell lymphoma cells were purchased from American Type Culture Collection (ATCC, Manassas, VA) and maintained via ATCC protocol.

Dendritic cells preparation was performed as previously described and modified [4]. Bone marrow–derived murine dendritic cells were generated by culturing bone marrow cells from the femur and tibiae of BALB/c mice at a starting concentration of 1×10^6^ cells/mL in RPMI-1640/10% fetal bovine serum (FBS; Invitrogen, Carlsbad, California) supplemented with 30 ng/mL of recombinant murine granulocyte macrophage colony-stimulating factor (mGM-CSF; PeproTech, Rocky Hill, NJ) and 3 ng/mL interleukin 4 (mIL-4; PeproTech, Rocky Hill, NJ). Fresh medium supplemented with GM-CSF plus IL-4 was added on day 3, and all of the loosely adherent cells were transferred to Petri dishes on day 6. 2 days later, nonadherent cells and loosely adherent dendritic cells were harvested, washed with PBS.

### Immune-magnetic beads isolation of MDSCs

Mouse spleen was grinded and red blood cells were eliminated by Tris-NH_4_Cl. Nucleated cells were resuspended in MACS buffer (pH7.2 PBS, 0.5% BSA, 2mM EDTA) at 1×10^8^ cells per 0.3mL. Cells were incubated with non-specific blocking buffer and magnetic beads conjugated Gr-1antibody at 10°C for 15 minutes. Gr-1^+^ cells were collected by magnetic column attraction. Purified cells were then analyzed by flow cytometry for their immunophenotype.

### Flow cytometry analysis

Lineage staining of bone marrow and spleen was performed with fluorescent conjugated antibodies against Gr1, CD11b, CD11c, Ia, CD31, Ter119, CD14, F4/80, CD3, CD4, CD8, B220, B7-1, B7-2 and DX5 (eBiosciences, San Diego, CA). Other antibodies used for flow cytometry include: CD34, Trail, c-kit, Flk-1, Sca-1, CD40 and CD54 (BD Biosciences, Franklin Lakes, NJ). Annexin V and PI were used for apoptosis analysis. Flow cytometry utilized Cytomics FC500 (Beckman Coulter Inc. Indianapolis IN), LSRII or FACSAria II, respectively (BD Biosciences). Flow cytometry data was analyzed with Kaluza, Diva, or FlowJo software. Cells were first gated with forward scatter (FSC) and side scatter (SSC) to exclude debris (small values <50 on FSC). The gated cells were then analyzed for the specific fluorescent channels with which the stained antibodies were conjugated. The results were represented with 2-D graphs as shown in the Figures section.

DCs were labeled as previously described [32] with carboxyfluorescein diacetate succinimidyl ester (CFSE) prior to the intratumoral injection. Briefly, DCs were resuspended in PBS (20×10^6^/ml) and an equal volume of CFSE (5μM in PBS) was added. After 10 min at room temperature, an equal volume of FBS was added for quenching. After one minute at 4°C, cells were washed with HBSS. 5×10^6^ CFSE-labeled DCs were intratumorally injected into each mouse.

### B-cell lymphoma mouse model and In vivo effects of gemcitabine and DCs

BALB/c mice were inoculated subcutaneously with 2x10^5^ A20 lymphoma cells, palpable subcutaneous tumor foci were detected under the skin within fourteen days. Large tumor blocks with a minimal volume of 700–1,000 mm^3^ were usually detected twenty-seven to thirty days after inoculation. The mice were then injected intraperitoneally with a single dose of 120 mg/kg of gemcitabine (Eli Lilly, Indianapolis, IN), or intratumorally with 5x10^5^ cultured DCs after 48 hours, or injected with both gemcitabine and DCs. Control mice received saline only. On selected days after treatment, spleen cells were isolated and the single cell suspensions were subjected to flow cytometry. Tumors volumes were estimated using the formula: (π x long axis x short axis x short axis) / 6. Each control or experimental group had five to ten mice. Mice survival was observed, recorded and represented by Kaplan-Meier survival curves. Mice with certain treatments survived beyond the endpoint (120 days), and were euthanized with CO_2_ followed by cervical dislocation.

### *In vitro* cytokine release assay

Spleen cells were harvested from the lymphoma bearing mice seven days after intra-tumoral injection of normal saline, DCs, or co-injection of both DCs and MDSCs. 2 × 10^6^ splenocytes were cocultured with 2 × 10^5^ A20 cells (25 mg/L mitomycin C treated for 30min) in 1 mL RPMI1640 medium containing 10% FCS in 24-well tissue culture plates. After 48 hours, cell-free supernatant were collected for measurement of murine IFNγ release by standard ELISA according to the manufacturer’s recommendations (eBioscience), which is reported as mean ± SD of triplicate samples.

### Cytotoxicity assay

Spleens were collected from mice and 5 x 10^6^ spleen cells were re-stimulated by co-culture with mitomycin C (25 mg/L for 30min)-treated A20 cells (3 x 10^5^) and 50 IU/mL murine interleukin 2 (mIL-2; PeproTech, Rocky Hill, NJ) for 5 days. After re-stimulation, target cells (2 x 10^4^/well) were cultured with re-stimulated splenocytes at various ratios in 96-well, round-bottomed plates (200 μL/well) for 6 hours at 37°C. After centrifugation for 10 min at 250g, 100 μL of supernatant from triplicate cultures were collected and the released lactate dehydrogenase (LDH) from target cells was measured in vitro using the CytoTox 96 Non-Radioactive Cytotoxicity Assay Kit (Promega, Madison, WI), according to the manufacturer’s protocol.

### Immunofluorescent staining

For immunofluorescence staining, antibodies against DX5-PE and NKG2D-FITC were purchased from eBiosciences. Tissues were fixed in 4% paraformaldehyde overnight, 30% sucrose incubation for 48 hours, and followed by frozen sectioning (3 μm). Slides were then pre-blocked with serum and stained with DX5 and NKG2D for one hour. Nucleuses were stained with DAPI and the slides were visualized with laser scanning confocal microscope (Zeiss LSM710).

### NK cells and T cells depletion

NK cell depletion was achieved using intraperitoneal injection of 300 mg of anti-asialo-GM1 antibody (WAKO Chemicals USA Inc, Richmond, VA) or isotype control Ab on day 24, 26, 28, 32 and day 35 (DC intratumoral injection is day 32). CD4^+^ and CD8^+^ T cells were individually depleted by intraperitoneal injection of 100 mg of anti-CD4 (GK1.5), or anti-CD8 (TIB105) mAbs (self-generated hybridomas) on day 24, 26, 28, 32 and 35 [33,34]. The efficiency of NK cells and T cells depletion was validated by flow cytometry analysis of splenocytes using anti-DX5, anti-CD4 and anti-CD8 mAbs. In all cases, >98% of the targeted cells subset was specifically depleted (data not shown).

### Data analysis

Kaplan-Meier survival curves were generated with GraphPad Prism 5 (GraphPad Software Inc, La Jolla, CA). Flow cytometry data were analyzed with CXP Analysis (Beckman Coulter Inc.), Diva (BD Biosciences) and FlowJo software (Tree Star Inc, Ashland, OR). Statistical analysis including the student t-test of the flow cytometry data, the log-rank analysis of the survival curve was performed with Excel (Microsoft Inc, Seattle, WA), GraphPad Prism 5, and SAS 9.1 (SAS Institute Inc. Cary, NC). Statistical data with replicates were represented by the average values and the standard deviation.

## Results

### Treatment of B-cell large size lymphoma in mice with either gemcitabine or cultured DCs alone failed to prevent tumor growth

**Fig 1:**
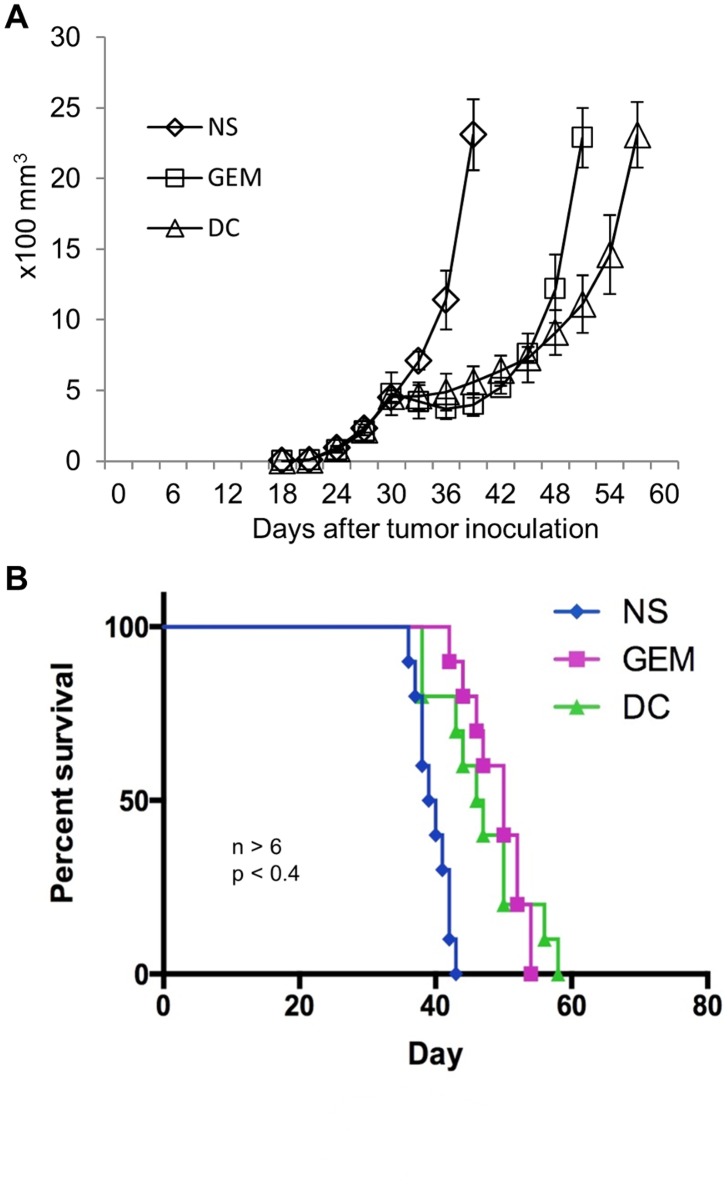
Gemcitabine chemotherapy or intratumoral injection of DCs failed to completely inhibit the growth of A20 cell induced large lymphoma on mice. **A,** tumor growth measurement on lymphoma-bearing mice injected with normal saline (NS), Gemcitabine (GEM), or DCs (DC) on day 30. Tumor volume was estimated by the formula: π x long axis x short axis x short axis) / 6. Each data point was represented by the average value of tumors from five mice and the corresponding standard deviation. **B,** Kaplan Meirer survival curve of mice injected with NS, GEM, or DC.

Chemotherapeutic reagent gemcitabine is routinely applied for clinical lymphoma treatment. To examine the inhibitory effect of gemcitabine on inhibition of large lymphoma growth, we intraperitoneally injected 120 mg/kg gemcitabine into A20 cell inoculated large lymphoma on BALB/c mice at day 30 after inoculation. As control treatment, we injected equal volume of normal saline into another group of tumor-bearing mice as well. Tumor developed from the normal saline treated mice continued to grow as large as 2,500 mm^3^, and the mice soon died within two weeks after saline injection (Fig 1A and 1B). For mice injected with gemcitabine, we observed that the tumor blocks softened; their sizes slightly reduced two days after gemcitabine treatment and maintained the same size for approximately additional ten days. This indicated that gemcitabine restricted tumor growth by eliminating some of the tumor cells. However, starting from the twelfth day since gemcitabine injection, tumor blocks continued to grow beyond control and became much larger in size. The mice eventually died, about twelve days later than those in the normal saline treatment group (Fig 1A and 1B).

Cancer vaccination with tumor cell released DAMPs-activated DCs has been shown to induce tumor regression in cancer patients [8]. However, recent clinical trials of vaccination with cultured, lymphoma DAMPs-activated DCs failed to demonstrate the expected, desired results [9]. It is possible that tumor antigen activated, *in vitro* matured DCs may not be as efficient as *in vivo* matured DCs in either function or quantity. To improve the potential efficacy of the DC mediated immunotherapy, A20 cell inoculated mice were intratumorally injected with inactivated, immature DCs. These DCs could be activated by lymphoma antigen onsite, will migrate to and mature within lymphatic organs, and develop into lymphoma antigen pulsed mature DCs *in vivo*. Intratumoral injection of DCs into lymphoma inoculated mice demonstrated a response that was similar to the gemcitabine treatment: tumors grew slowly and the sizes became smaller, but the tumor eventually grew out of control and the mice died two weeks later (Fig 1A and 1B). Thus, treatment of large size lymphoma with either chemotherapeutic gemcitabine, or DCs therapy alone, can only modestly restrict tumor growth and slightly extend the survival of the lymphoma-bearing mice. Neither gemcitabine nor DCs mediated immune vaccination completely inhibited tumor cell growth or rescued these mice died of tumor progression.

### Accumulation of MDSCs in the spleen of lymphoma-bearing mice

**Fig 2:**
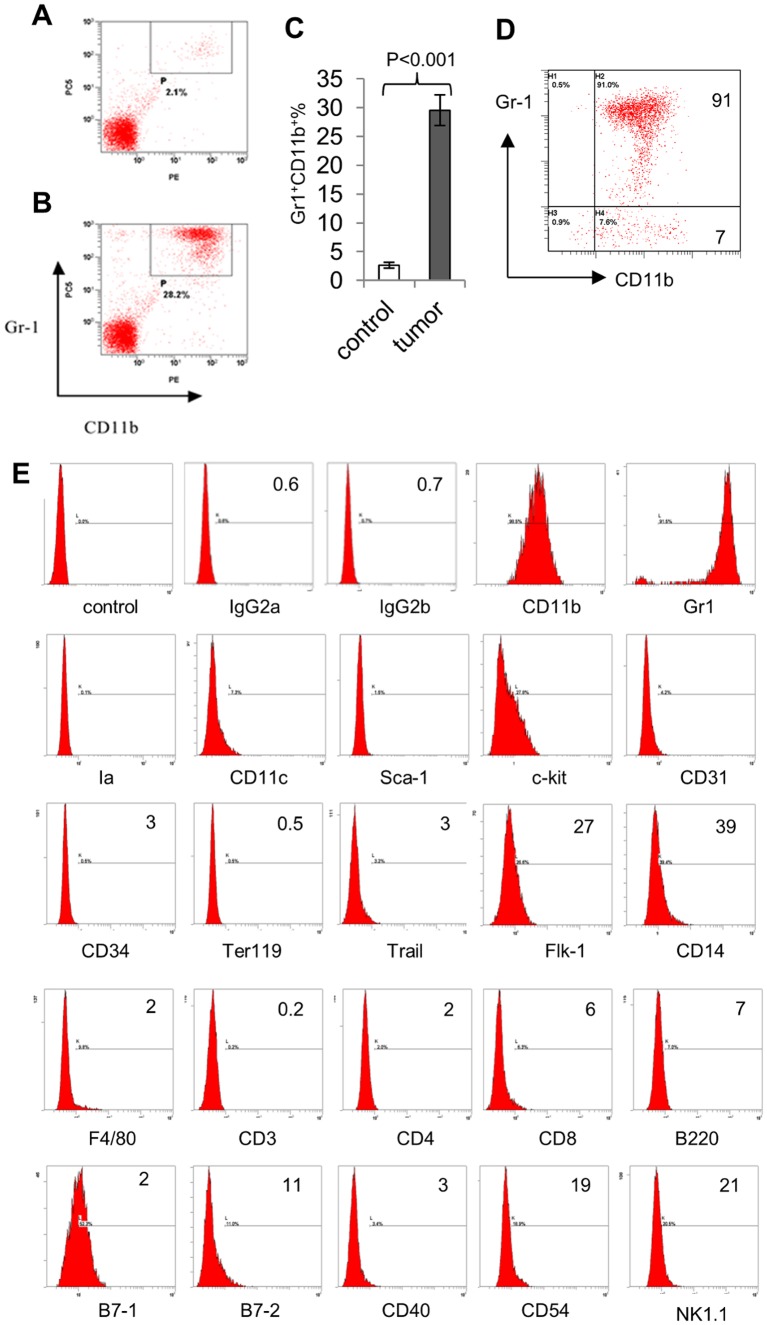
The percentage of Gr-1^+^CD11b^+^ cells in the spleens of normal mice A, and mice bearing A20 cell induced large lymphoma (30 days) B, summary with statistical analysis of average and standard deviation were shown in C (n = 3). Flow cytometry analysis of the immunophenotype of magnetic-beads isolated Gr1+CD11b+ MDSCs sorted from the spleens of tumor-bearing mice. **D,** purity check on Gr1^+^CD11b^+^ MDSCs after sorting. **E,** flow cytometry analysis of the immunophenotype of isolated Gr-1^+^CD11b^+^ MDSCs; numbers represent the percentages of positively antibody-stained cells.

We examined the mice thirty days after A20 cells inoculation. We found the sizes and weights of their spleen were comparable to the control mice. But the cellular components of the spleen were quite distinct. There were significantly increased percentages (22.3% -35.8%, n> = 10) of Gr-1^+^ and CD11b^+^ MDSCs in the spleen of the lymphoma-bearing mice. In contrast, the percentages of MDSCs in the spleen of the control mice were merely 2.2% on average (Fig 2A–2C). Because the sizes and weights of the spleen in tumor-bearing mice were slightly larger than those of the control mice, the quantity of the Gr-1^+^CD11b^+^ MDSCs in the spleen of the tumor-bearing mice was approximately ten times more than that in the spleen of the control mice.

To further analyze the immunophenotype of MDSCs, spleen cells harvested from both control and tumor-bearing mice were labeled with magnetic beads or florescent dye conjugated Gr-1 antibody and MDSCs were collected by magnetic column separation (MACS) or florescence activated cell sorting (FACS). Flow cytometry analysis of the purified MDSCs confirmed that more than 90% of cells were Gr-1^+^CD11b^+^, indicating that the sorted cells were enriched with MDSCs (Fig 2D). These Gr-1^+^CD11b^+^ MDSCs expressed cell surface antigens c-kit, B7-1, Flk-1 and CD14 at intermediate levels (Fig 2E).

### Gemcitabine induced increased apoptosis of MDSCs *in vitro* and *in vivo*

**Fig 3:**
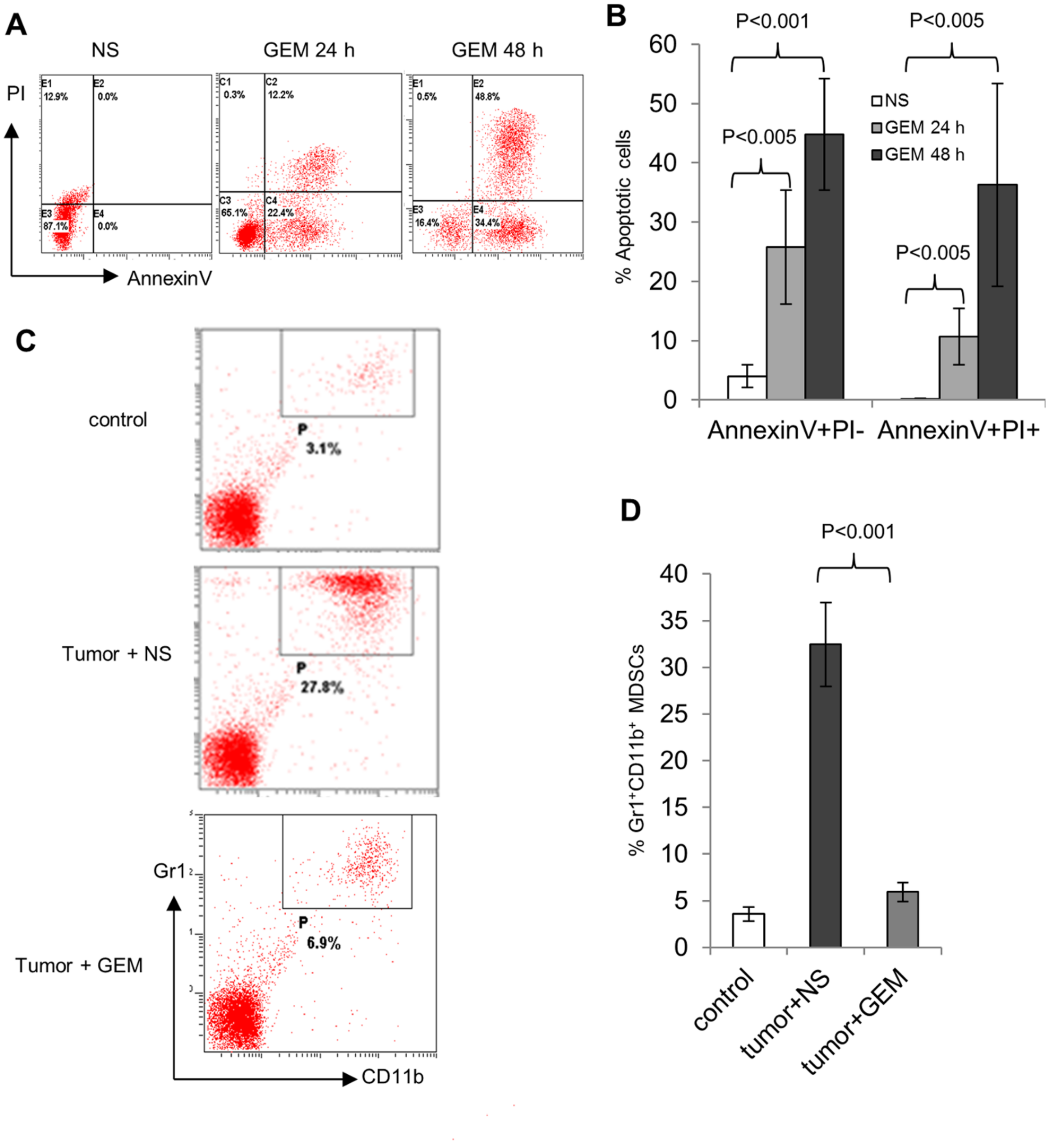
Gemcitabine induced apoptosis of in vitro cultured MDSCs. **A,** MDSCs isolated from tumor bearing mice were incubated in culture with normal saline (NS) as controls; Gemcitabine (100 ug/ml) for 24 and 48 hours, stained with Annexin V and propidium iodide (PI), and analyzed by flow cytometry. **B,** results from three replicates were summarized and represented with the average values and their standard deviations, and statistical p-values for student t-test pair-wise comparison were included. Gemcitabine reduced the number of MDSCs in the spleen of tumor-bearing mice. **C,** flow cytometry analysis of Gr1^+^CD11b^+^ MDSCs in the spleen of non-tumor normal mice (control), mice bearing A20 cell induced large lymphoma treated with normal saline (NS), or Gemcitabine (GEM). **D,** summary of the results from C with three replicates was represented by the average values and the standard deviations, and statistical p-values for student t-test pair-wise comparison were included.

Because of the immunosuppressive effects induced by MDSCs, the significantly increased MDSCs in the spleen may account for the poor anti-tumor effects of the A20-induced lymphoma-bearing mice after vaccination with DCs. Thus, elimination of these MDSCs may reduce the immunosuppressive effects and potentially improve the anti-tumor immune responses of the cancer immunotherapy. We next performed the experiment to examine whether gemcitabine induces the increased apoptosis in MDSCs. MACS purified MDSCs were placed in culture and incubated with normal saline or gemcitabine at 10 μg/L. After 24 to 48 hours cultured MDSCs were labeled with fluorescein conjugated Annexin-V antibody and stained with propidium iodide (PI). In striking contrast to the control treatment, flow cytometry analysis showed that more than 34% and 83% of MDSCs were Annexin-V positive after 24 or 48 hours of treatment, respectively (Fig 3A and 3B). Thus, gemcitabine induces significantly increased apoptosis in cultured MDSCs after 48 hours of treatment.

We also evaluate the effect of gemcitabine on spleen MDSCs from A20-induced lymphoma-bearing mice. Thirty days after tumor cells inoculation, mice were intraperitoneally injected with 120mg/kg of gemcitabine, or the same volume of normal saline as control treatment. Spleen cells were collected from these mice two days later, labeled with Gr-1 and CD11b antibodies, and subject to flow cytometry analysis. As shown in Fig 3C and 3D, A20 lymphoma cells induced to generate more than 30% of Gr1^+^CD11b^+^ MDSCs in the spleen of the tumor-bearing mice injected with normal saline. In contrast, mice injected with gemcitabine markedly reduced the percentage of MDSCs down to 7%, which was slightly higher than the MDSC percentage in non-tumor-bearing normal mice. Thus, chemotherapeutic dosage of gemcitabine quickly eliminated the majority of spleen MDSCs both *in vitro* and *in vivo*.

### Gemcitabine treated A20 cells *in vivo* promoted the maturation of DCs

**Fig 4:**
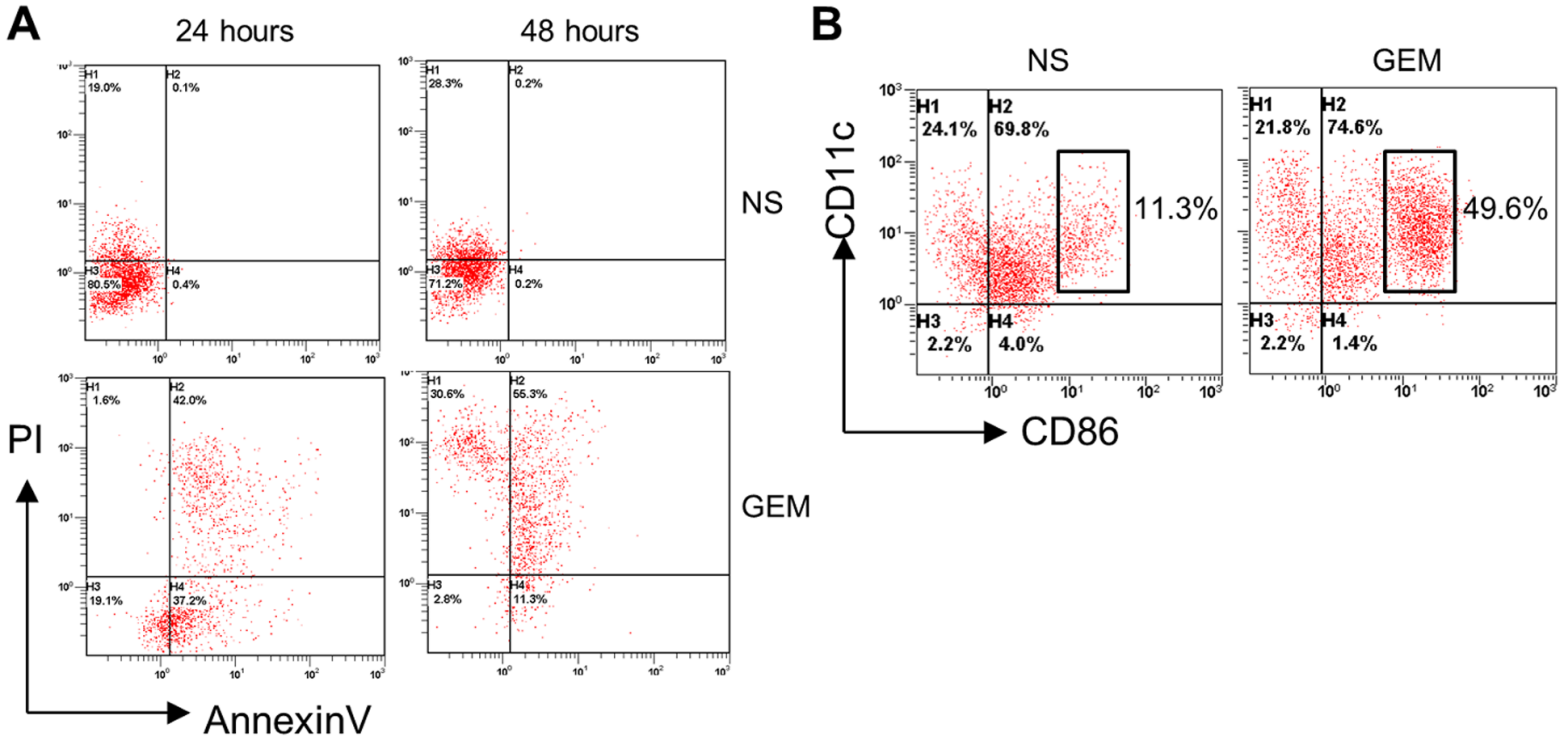
Treatment of A20 lymphoma cells with gemcitabine increased apoptosis and promoted dendritic cell maturation. **A,** flow cytometry analysis of normal saline (NS) or gemcitabine (GEM) treated A20 cells cultured for 24 and 48 hours for annexinV and propidium iodide (PI) staining. **B,** flow cytometry analysis of dendritic cell maturation induced by NS or GEM treated A20 cells (48 hours) for the expression of CD11c and CD86.

**Fig 5:**
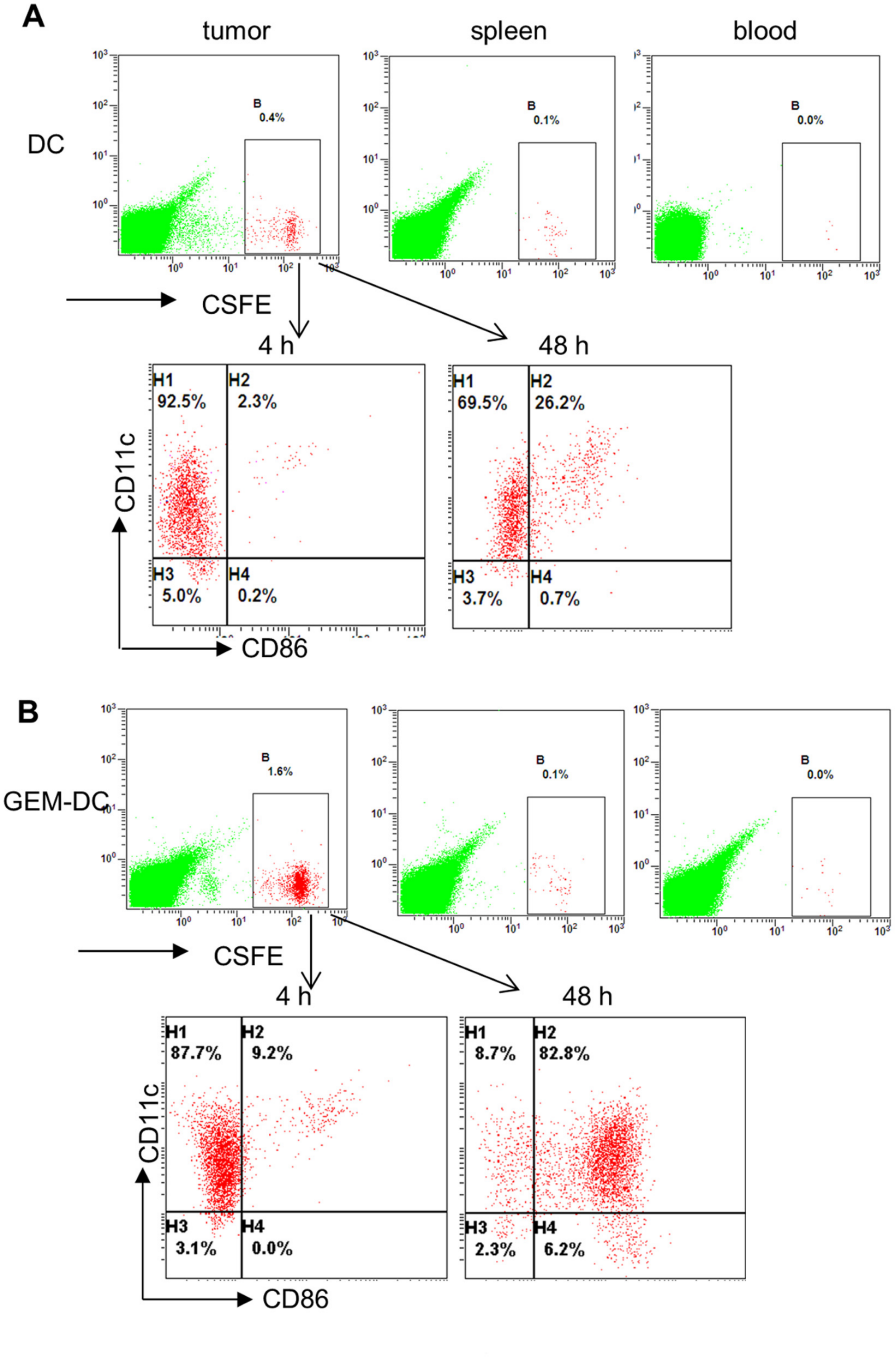
Lymphoma bearing mice treated with gemcitabine and intratumoral injection of DCs promoted dendritic cell maturation *in vivo*. CSFE labeled DCs were injected into A20 cells induced lymphoma in mice treated with DCs alone **A,** or DCs and gemcitabine (GEM-DC) **B,** and DCs were collected from lymphoma tissue, blood or spleen, and analyzed by flow cytometry for CSFE and DC maturation (CD11c and CD86).

It is conceived that intra-tumoral injected immature DCs will be activated *in vivo* by lymphoma released DAMPs and induced to differentiate within lymphatic organs, and will develop into functional DCs. Inoculated A20 lymphoma cells from mice that were treated with normal saline (NS) for 48 hours did induced modest DC maturation, as shown in Fig 4B (left panel; quadrants were based on the total spleen cells from NS treated, non-tumor bearing mice). We hypothesized that gemcitabine induced apoptosis of A20 cells *in vivo* generated the lymphoma DAMPs that were even more efficient on promoting the development of DCs. To examine this, mice inoculated with A20 cells were injected with gemcitabine and the lymphoma cells were collected 24 hours or 48 hours later. As shown in Fig 4A, gemcitabine treatment of A20 lymphoma cells induced increased apoptosis in mice; the percentages of apoptotic cells further increased 48 hours after gemcitabine injection. Bone marrow derived immature DCs were then cultured with these lymphoma cells from NS or gemcitabine treated, A20 cell inoculated mice. Flow cytometry analysis demonstrated that gemcitabine treated, apoptotic A20 cells markedly increased the percentages of mature DCs with cell surface expression of CD86 (Fig 4B). To further confirm the DCs activation *in vivo*, we intratumorally injected labeled bone marrow derived immature DCs with carboxyfluorescein diacetate succinimidyl ester (CFSE) into mice bearing lymphoma treated with or without gemcitabine. Cells from tumors, spleen and blood were prepared for flow cytometry analysis. As shown in Fig 5, CFSE labeled DCs primarily accumulated in lymphoma tumors rather than in other hematopoietic organs. Mice treated with gemcitabine and injected with DCs demonstrated a significant increase (p<0.01) in the percentage of CFSE labeled DCs in comparison to mice injected with DCs only (Fig 5A). Flow cytometry analysis of these CFSE labeled DCs 48 hours after intratumoral injection revealed that they were primarily mature, activated DCs with CD11c and CD86 expression (Fig 5B). The relative increase of CFSE labeled DCs in mice treated with gemcitabine and injected with DCs was likely due to the elimination of some lymphoma cells by gemcitabine. Compared with NS treated lymphoma cells, gemcitabine treated A20 cells induced increased apoptosis, provided the processed, endogenous lymphoma released DAMPs, and significantly promoted the differentiation and maturation of DCs.

### Combination of gemcitabine with DC mediated tumor vaccination significantly reduced tumor size and extended mice survival

**Fig 6:**
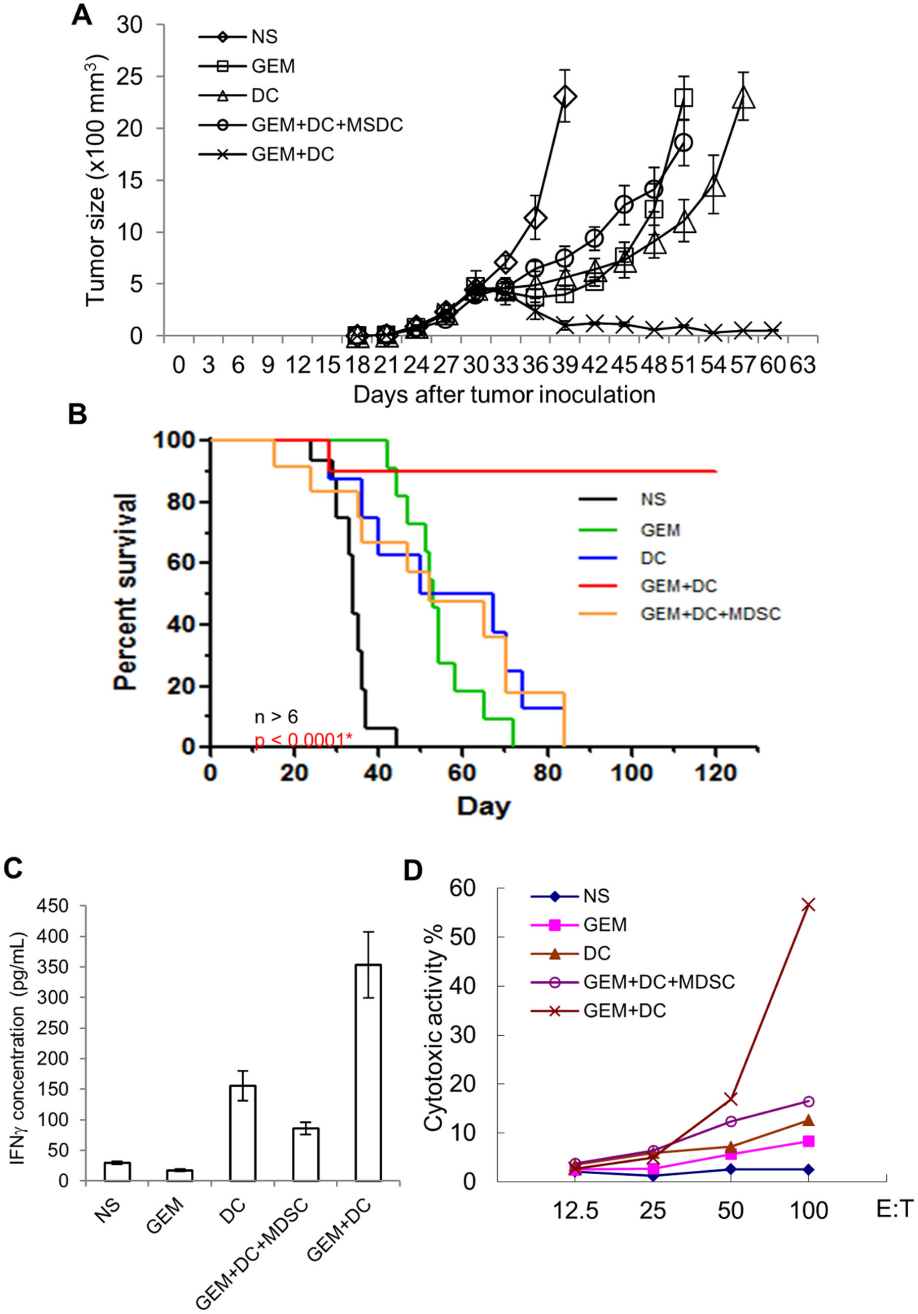
Combination of gemcitabine chemotherapy with DC cancer vaccination efficiently inhibited the growth of A20 cell induced large lymphoma and significantly promoted the survival of the tumor bearing mice. **A,** tumor volume sizes on mice injected with normal saline (NS) on day 30, Gemcitabine on day 30 combined with intratumoral injection of DCs on day 32 (GEM+DC), or Gemcitabine on day 30, DCs and MDSCs on day 32 isolated from the spleen of tumor bearing mice (GEM+DC+MDSC). Each data point was represented by the average value of the tumors from five animals and the corresponding standard deviation. **B,** Kaplan Meirer survival curve of mice injected with NS, GEM+DC, or GEM+DC+MDSC. Mice that survived beyond 120 days were euthanized. **C,** ELISA of the secreted IFNγ in the supernatant of cultured spleen cells from lymphoma bearing mice re-stimulated with mytomycin C treated A20 cells for 48 hours. **D,** Cytotoxic activity assays of the spleen cells from lymphoma bearing mice re-stimulated with mytomycin C treated A20 cells and murine interleukin 2 for five days, followed by six-hour co-culture of these re-stimulated spleen cells (effector cells) with A20 cells (target cells) at different ratios (E:T ratio); supernatant LDH released from the target cells were measured, normalized and represented as percentages.

Gemcitabine not only inhibited tumor growth by inducing apoptosis, promoted differentiation of DCs, but also eliminated the majority of MDSCs in the spleen of the large lymphoma bearing mice. We next performed the experiment to examine whether combination of gemcitabine with immune vaccination could significantly improve the efficacy of either treatment for large size lymphoma. Mice were first inoculated subcutaneously with A20 cells to induce B-cell lymphoma. Thirty days later, mice were injected with normal saline for control treatment and developed subcutaneous tumor foci. As expected, the tumor continued to grow into large tumor block which eventually caused the death of all animals (Fig 6A and 6B). Mice injected with 120 mg/kg of gemcitabine were also intra-tumorally injected with cultured, activated DCs 48 hours later. Distinct from the mice injected with normal saline, mice treated with both gemcitabine and DCs demonstrated decreased size of tumor blocks and they continued to shrink for thirty days during observation period. The mice survived significantly longer than the control treatment group and 85% of these mice survived longer than ninety days as well. To confirm that the improved treatment efficacy was due to the gemcitabine caused elimination of MSDCs, gemcitabine and DC treated lymphoma bearing mice were also injected with 5x10^6^ purified MDSCs. The tumor of these mice grew slowly than the mice with normal saline treatment. They survived longer as well. But similar to the mice treated with gemcitabine or DCs alone, the tumor grew into larger blocks a few weeks later and the mice eventually died. Because intra-tumorally injected DCs were efficiently activated by the gemcitabine induced apoptosis of A20 lymphoma cells, these activated DCs would stimulate spleen lymphocytes to produce cytokines and induce the tumor specific cytotoxic activity. To confirm this, we measured the ability of the spleen cells to produce interferon gamma (IFNγ) from A20 inoculated lymphoma bearing mice with different treatments. As shown in Fig 6C, spleen cells from the lymphoma bearing mice with intra-tumorally injected DCs demonstrated a fourfold increase in producing IFNγ, when compared with the spleen cells from the normal saline or gemcitabine treated mice; the spleen cells from the lymphoma bearing mice that were sequentially treated with gemcitabine and DCs showed a tenfold increase in the stimulation of IFNγ production; co-injection of MDSCs and DCs after gemcitabine treatment reversed the increased the production of IFNγ at a level less than the lymphoma bearing mice treated with DCs alone. We also examined the cytotoxic activity in the lymphoma bearing mice with different treatments. Similarly, spleen cells from mice that were treated with either gemcitabine or DCs alone, demonstrated slight increase in cytotoxic activity compared with the NS treated mice. Treatment of lymphoma bearing mice with both gemcitabine and DCs significantly increased the cytotoxic activity of the spleen (Fig 6D). Thus, treatment of lymphoma bearing mice with DCs alone modestly increased the ability of their spleen cells for cytokine production and cytotoxic activity; treatment of these mice with both gemcitabine and DCs markedly induced the IFNγ cells and their lymphoma specific cytotoxic activity within the spleen cells.

### Gemcitabine combined with DCs vaccination induced activation of NK cells and both NK cells and T lymphocytes are required for inhibition of tumor growth

**Fig 7:**
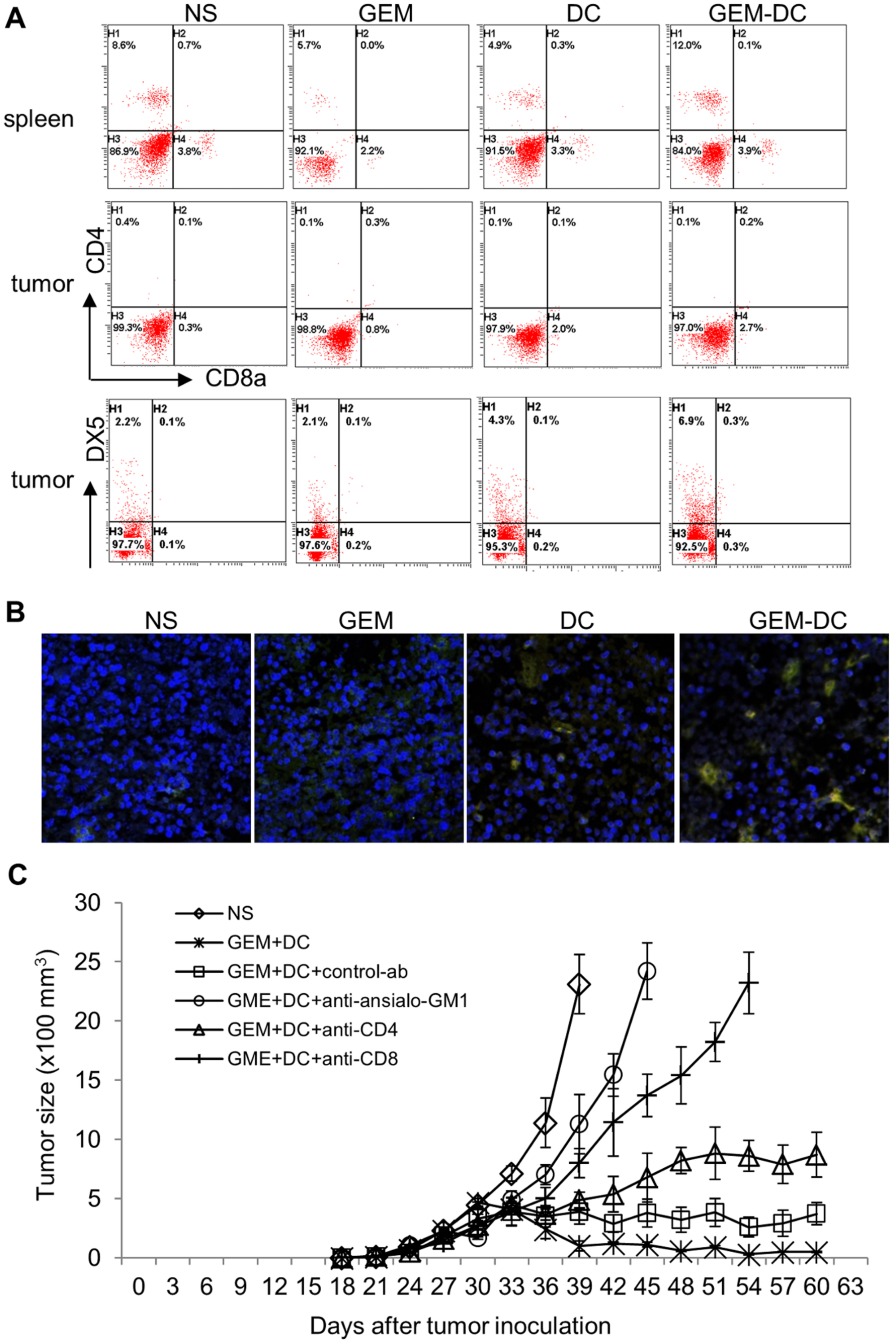
NK cells appeared to be the major effector cells to inhibit tumor growth. Mice bearing A20 cells induced lymphoma were treated with normal saline (NS), gemcitabine (GEM), intratumorally injected with DCs, or the combination of gemcitabine and DC injection (GEM-DC). Tumor or spleen tissues were collected and analyzed by **A,** flow cytometry NK cells (DX5) and T lymphocyte specific antigens (CD4 and CD8). **B,** Immunofluorescent staining of lymphoma tissues with NK specific antibodies (DX5 red; NKG2D green), and nucleus were stained with DAPI (blue). **C,** Tumor volume sizes on mice injected with normal saline (NS), Gemcitabine combined with intratumoral injection of DCs (GEM+DC), or pre-treated with injection of anti-asialo-GM1, anti-CD4, anti-CD8 or IgG and gemcitabine combined with intratumoral injection of DCs (GEM+DC+anti-asialo-GM1, GEM+DC+anti-CD4, GEM+DC+anti-CD8 or GEM+DC+control-ab). Each data point was represented by the average value of tumors from five mice and the corresponding standard deviation.

DC induced efficient generation of CD8^+^ CTLs, CD4^+^ T helper cells and NK cells [35]. We next sought to find out which are the primary intermediate effector cells for tumor elimination. Five days after treatment of mice with normal saline, gemcitabine or/and intratumoral injection of DCs, lymphoma tissues were collected for flow cytometry and immunofluorscenece analyses. As shown in Fig 7A, the percentage of CD4^+^, CD8^+^ cells was comparable in the tumor samples within all four treatments. In contrast, the percentage of DX5^+^ cells increased significantly in the tumor sample of mice treated with gemcitabine and injected with DCs (p<0.01). To further confirm the increased presence of NK cells in gemcitabine treated and DCs injected mice, we performed immunohistochemistry staining of lymphoma tissues with both anti-DX5 (red) and anti-NKG2D (green) antibodies to label activated NK cells. Fig 7B demonstrated the increased co-localization of both antigens (yellow), which confirmed the increased number of NK cells in the tumor tissues of mice treated with both gemcitabine and injected DCs. This finding indicated that it was mainly the activated NK cells accumulating in the A20 cells induced lymphoma elimination.

Although more NK cells accumulated in the tumors than T cells when they were examined, the kinetics of migration of T and NK cells may be different. To find out whether NK cells or T cells are the major effector cells required for the DC initiated anti-tumor immunity, we depleted NK cells with anti-asialo-GM1 antibody during the combination treatment of the A20 cells induced lymphoma in mice [33]. We also depleted CD4^+^ and CD8^+^ T cells with anti-CD4 and anti-CD8 monoclonal antibodies in parallel experiments. In contrast to the control antibody treatment, which served as a non-specific control and demonstrated the tumor growth curve similar to that of the combined treatment with gemcitabine and injected DCs, co-injection of anti-asialo-GM1 antibody, gemcitabine and DCs failed to inhibit tumor growth (Fig 7C). Injection of anti-CD4 antibody, gemcitabine and DCs did not inhibit tumor growth, but injection of anti-CD8 antibody greatly reduced tumor sizes. CD8^+^ T cells depletion resulted in delayed tumor growth compared with the tumor growth in the NK cells depletion experiment (Fig 7C). Thus, gemcitabine combined with DCs vaccination induced accumulation of NK cells in tumor; and both NK cells and CD8^+^ T cells appeared to be the major effector cells mediating tumor cell elimination *in vivo*. In conclusion, combination of chemotherapeutic gemcitabine and immune vaccination with DCs efficiently removed the lymphoma induced accumulation of MDSCs, and synergistically induced the IFNr production and activation of NK cells and CD8^+^ T cells, and thus enhanced anti-tumor immunity and improved the survival of the lymphoma bearing mice.

## Discussion

Lymphoma is among the cancer category that is sensitive to the DC mediated immunotherapy [36,37,38,39]. Recently, we and others have carried out a series of basic and clinic experiments, confirming that subcutaneous inoculation with lymphoma antigen pulsed DCs can induce anti-lymphoma immune response. Intra-tumoral injection of non-antigen pulsed semi-mature DCs produced even better effect on inhibition of tumor growth in a short period of time. This is probably due to the better maintenance of the antigen processing and presenting ability of the non-pulsed DCs, the more efficient, on-site tumor antigen acquisition and presentation that is released from the apoptosis of the tumor cells, and the more efficient activation of the downstream cytokine secreting Th1 and cytotoxic T-lymphocytes and NK cells. These DC mediated immune therapies have been proposed to treat patients with relapsed or refractory lymphoma, also due to the fact that the relapsed or refractory lymphoma tumors are often palpable and it is possible for performing the accurate intra-tumoral injection of DCs.

In this work, we started with the intra-tumoral DC immunotherapy for the treatment of the relapsed or refractory lymphoma induced by A20 cell inoculation in mice. Although we observed that the tumor sizes reduced to half of their original sizes 4–10 days after treatment, the tumors did not disappear; instead, they restored rapid growth 2–4 weeks later. Therefore, intra-tumoral DC immunotherapy alone is not sufficient enough for the effective treatment of the relapsed or refractory large lymphoma. Alternative strategies are required to improve the current efficacy of DC based immunotherapy.

It is believed that the inefficiency of DC mediated immunotherapy was partially due to the tumor cell induced accumulation of MDSCs in patients. MDSCs accumulated in the spleen of tumor bearing mice and surrounding the tumor tissues to inhibit lymphocyte activation and promote tumor angiogenesis and tumor growth. Removal of MDSCs could alleviate the tumor-induced, MDSC-mediated immune suppression, enhance the immune response, and significantly increase the effects of cancer vaccination in suppression of mouse tumor growth in vivo [13]. But feasible clinic approaches to eliminate MDSCs from patients are yet to be defined and examined. It has been reported that the all-trans retinoic acid (ATRA) and IL-4 induced MDSCs to differentiate into mature myeloid cells and enhanced the efficacy of tumor vaccine [27]. Our experiments demonstrated that ATRA, IL-4 or LPS failed to induce *in vitro* differentiation of isolated Ly6C^+^ MDSCs, nor did they enhance the anti-tumor effects of DC vaccine (unpublished results).

Gemcitabine has been used to treat some relapsed or refractory lymphoma. Gemcitabine not only inhibits the growth of tumor cells by increased tumor cell apoptosis induction, it also eliminates existing MDSCs from the body by inducing increased apoptosis. In our study, we demonstrated that MDSCs accumulated in the spleen of the A20 lymphoma bearing mice. The percentage or number of MDSCs increased up to ten times higher than the control mice. 24–48 hours incubation of 10 mg/L gemcitabine with FACS isolated MDSCs in cell culture induced markedly increased apoptosis in MDSCs. Injection of 120mg/kg gemcitabine into A20 lymphoma-bearing mice eliminated the majority of Gr-1^+^CD11b^+^ MDSCs in the spleen in two days. Gemcitabine treatment or intra-tumoral DC injection alone inhibited the tumor growth and prolonged their survival time of the lymphoma bearing mice. But neither can completely eliminate tumor cells or rescue the tumor bearing mice. The tumor size became much smaller a few weeks after gemcitabine treatment. But the small number of tumor cells that were resistant to gemcitabine eventually restored tumor growth. Intra-tumoral injection of inactivated DCs 48 hours after gemcitabine treatment could induce DCs maturation by in vivo lymphoma antigen presentation which was generated by gemcitabine treatment. This in turn induced the cytokine secreting cells and activated the NK and CD8^+^ T cells. This improved DC mediated immunotherapy, combined with gemcitabine chemotherapy, was believed to efficiently eliminate the small number of residual gemcitabine resistant lymphoma cells because gemcitabine also selectively removed the immune-suppressive MDSCs without causing damages on other immune cells such as T-lymphocytes and natural killer cells. 5-fluorouracil (5-FU) is another known chemotherapeutic agent that is capable of eliminating MDSCs in vivo. Unfortunately 5-FU has not been approved for the treatment of lymphoma. In our study, combination of both the gemcitabine chemotherapy and the DC mediated immunotherapy on A20 cell induced, large lymphoma bearing mice completely prevented the tumor growth and rescued >80% of the mice. Thus, our study provides an improved strategy by synergizing both the chemotherapeutic agent gemcitabine and the intra-tumoral delivered DC mediated immunotherapy to significantly enhance the efficacy of each individual treatment on some relapsed or refractory large lymphoma.
